# Water Extract of *Rhizoma Drynaria* Selectively Exerts Estrogenic Activities in Ovariectomized Rats and Estrogen Receptor-Positive Cells

**DOI:** 10.3389/fendo.2022.817146

**Published:** 2022-02-24

**Authors:** Liping Zhou, Ka-Ying Wong, Christina Chui-Wa Poon, Wenxuan Yu, Huihui Xiao, Chi-On Chan, Daniel Kam-Wah Mok, Man-Sau Wong

**Affiliations:** ^1^Cell Therapy Center, Xuanwu Hospital Capital Medical University, Beijing, China; ^2^Department of Applied Biology and Chemical Technology, The Hong Kong Polytechnic University, Hong Kong, Hong Kong SAR, China; ^3^Research Center for Chinese Medicine Innovation, The Hong Kong Polytechnic University, Kowloon, Hong Kong SAR, China; ^4^State Key Laboratory of Chinese Medicine and Molecular Pharmacology (Incubation), The Hong Kong Polytechnic University Shenzhen Research Institute, Shenzhen, China

**Keywords:** tissue selectivity, selective estrogen receptor modulators (SERMs), rhizoma drynaria, phytoestrogen, estrogen receptors, estrogenic activities

## Abstract

Our previous study demonstrated that the bone protective actions of herbal medicine *Rhizoma Drynariae* (Gusuibu, RD) were mainly mediated by flavonoid phytoestrogens *via* estrogen receptors, raising concerns about the safety of using RD as it may induce estrogen-like risk-benefit profile and interact with other ER ligands, such as selective estrogen receptor modulators (SERMs), when coadministered. The present study evaluated the estrogenic activities of RD and its potential interaction with tamoxifen, a SERM, in estrogen-sensitive tissues by using mature ovariectomized (OVX) rats and ER-positive cells. Similar to but weaker than tamoxifen, RD at its clinical dose dramatically ameliorated OVX-induced changes in bone and dopamine metabolism-related markers in OVX rats. However, tamoxifen, but not RD, induced uterotrophic effects. No significant alteration in mammary gland was observed in OVX rats treated with RD, which was different from the inhibitory actions of tamoxifen. The two-way ANOVA results indicated the interactions between RD and tamoxifen in the bone, brain, and uterus of OVX rats while RD did not alter their responses to tamoxifen. Our results demonstrate that RD selectively exerts estrogenic actions in a different manner from tamoxifen. Moreover, RD interacts with tamoxifen without altering its effects in OVX rats.

## Introduction

Hormone replacement therapy (HRT) carries considerable benefits for treatment of menopausal syndrome, like vasomotor symptoms and postmenopausal osteoporosis. However, HRT significantly increases the risk of breast cancer, endometrial cancer, and cardiovascular disease, making HRT a subject of argument (1). Selective estrogen receptor modulators (SERMs) act as either estrogen receptor (ER) antagonist or agonist depending on the tissue type and are clinically prescribed to postmenopausal women as an alternative to HRT. Tamoxifen is clinically used for treatment of breast cancer as it is an ER antagonist in breast. On the other hand, it exerts antiosteoporotic effects in menopausal women as an ER agonist in bone (2). However, due to undesirable uterine abnormalities, depression, and increased risk of endometrial cancer after tamoxifen administration, menopausal women try to seek help from alternative approaches (3).

Phytoestrogens derived from plants are also called natural estrogen and are the most popular alternative approach (4). They act in the same way as SERMs as they could activate ERs and exhibit various estrogenic and antiestrogenic effects in different tissues. As a main source of phytoestrogens, herbal medicines are vigorously promoted because of their perceived health benefits and minimal side effects (4). Indeed, the demands for herbal medicine for management of menopausal symptoms are increasing worldwide.

*Rhizoma drynariae* (*Gusuibu*, RD or GSB), the dried rhizome of perennial pteridophyte *Drynaria fortunei*, was first recorded in “Ben Cao Shi Yi” over 1,000 years ago and is recognized as a “kidney-tonifying” herb for bone-related disorders such as osteoporosis and bone fracture for many years in China. RD extract has been reported to mimic estrogen and increase bone formation in OVX mice and promote osteoblastic differentiation in pre-osteoblastic MC3T3-E1 cells (5). The major bioactive constituents of RD are demonstrated to be flavonoids, among which naringin and neoeriocitrin are the two richest flavonoids (6). Our previous works confirmed the antiosteoporotic activity of RD in preclinical model and further reported that total flavonoids of RD and naringin could mimic estrogen in stimulating cell proliferation and alkaline phosphatase (ALP) activity *via* activating ER in rat osteoblastic UMR106 cells (7, 8). These results suggest that RD contains phytoestrogens, which mediate the bone-protective effects of RD *via* ERs.

As more and more postmenopausal women are using phytoestrogens and phytoestrogen-containing herbal medicine for relieving their menopausal symptoms, concerns about their safety have been raised whether they will bring similar risk-benefit profile as estrogen and SERMs. Therefore, it is of particular importance to investigate whether these phytoestrogen-containing herbal medicines, like RD, could selectively induce estrogen-like activities in target tissues without inducing undesirable actions. Moreover, it will be crucial to determine if RD interacts with SERMs to either increase or decrease their pharmacological activities, which is of fundamental significance to the medical community and those postmenopausal women with breast cancer who simultaneously take supplements, like herbal medicines, together with their standard treatment.

Our current work defined and compared the possible estrogenic or antiestrogenic effects of RD with those of tamoxifen in the bone, brain, uterus, and breast by using both *in vivo* mature OVX rats and ER-positive cell lines. In addition, the potential interactions between RD and tamoxifen were also determined.

## Methods

### Preparation and Chemical Analysis of RD Extract

Dry aerial part of *rhizoma drynariae* was purchased from mainland China and authenticated by Dr. Chen Sibao at the State Key Laboratory of Chinese Medicine and Molecular Pharmacology (Incubation) in Shenzhen (No.: SZ2016HEP01). High-performance liquid chromatography (HPLC) assays were conducted to ensure that the quality of the raw herb fulfill the requirement of the China Pharmacopoeia and/or the Hong Kong Chinese Materia Medica Standards. Upon authentication, raw herb was delivered to Xi’an Pincredit Bio-tech Co., Ltd. for extract preparation. Briefly, RD was boiled in 10 volumes of water (v/w) for 20 min and for another 1 h in triplicate. The extract was obtained by filtration and dried under vacuum and stored at −20°C. The amount of naringin extract, the main active compound of RD, was quantified in RD extract by liquid chromatography-mass spectrometry (LC-MS).

### Animal Experiment and Sample Collection

All procedures involving animals in the present study were approved by the Hong Kong Polytechnic University Animal Subjects Ethics Sub-committee (ASESC Case: 15-16/31-ABCT-R-HMRF). Six-month-old female Sprague-Dawley rats were ovariectomized or sham-operated (Sham). After a 2-week recovery, the OVX rats were randomly subjected into 5 treatment groups (10 rats/group) and orally given double-distilled water (OVX), water suspension of 17ß-estradiol (E2, 1.0 mg/kg/day), RD (0.2 g/kg/day), tamoxifen (Tamo, 0.1 mg/kg/day), or the combined use of RD and tamoxifen for 3 months. During the whole treatment period, the animals were paired fed with phytoestrogen-free AIN-93M diet (composition was provided in **Supplementary Table S1**) to avoid the influence of phytoestrogens in the diet. At euthanasia, urine, serum, uterus, breast tissue, lumbar spine, and striatum were collected to evaluate the specific estrogenic parameters in each tissue.

### Biochemical Assay of Serum and Urine

Serum estradiol, follicle-stimulating hormone (FSH) and luteinizing hormone (LH), and osteocalcin (OCN), as well as urinary deoxypyridinoline (DPD) were measured by using EIA or ELISA commercial kits (EIA Estradiol Kit, CayMan; FSH and LH ELISA Kits, CloudClone, Katy, TX, USA; Osteocalcin ELISA Kit, Alfa Aesar, Royston, UK; MicroVue DPD EIA Kit, Quidel Corporation, San Diego, CA, USA).

### BMD and Micro-CT Analysis

Bone properties of the trabecular bone at the lumbar vertebra (L2–L5) were determined by micro-CT (μCT40, Scanco Medical, Wangen-Brüttisellen, Switzerland) as described in our previous study (9). The source energy selected for this study was 70 kVp and 114 μA with a resolution of 21 μm. Approximately 200 slices were done for each scan. Bone mineral density (BMD, mg HA/ccm) and bone morphometric properties, including bone volume over total volume (BV/TV), connectivity density (Conn.D, 1/mm^3^), trabecular bone number (Tb.N, mm^−1^), trabecular bone thickness (Tb.Th, mm), and trabecular bone separation (Tb.Sp, mm), were evaluated by contoured volume of interest images.

### Hematoxylin-Eosin Staining

Uterus and breast tissues were fixed in 4% paraformaldehyde for 6 h. Tissues were embedded in paraffin, and 8-µm-thick sections were produced for each sample after dehydration (Leica TP1020). Five sections from each sample were observed using ×400 (uterus) or ×100 (breast) magnification and photographed using a photoscope (Olympus BX51).

### Real-Time PCR Assay

Collected tissues were homogenized in Trizol reagent by using Precellys 24 homogenizer (Bertin, Fontaine, France). A total of 2.0 μg of total RNA was used to generate cDNA by using High-Capacity cDNA Reverse Transcription Kits (Applied Biosystems, Cheshire, UK) following the manufacturers’ instruction. Furthermore, 20 μl of PCR reaction system consisting of 1 μl cDNA, 0.4 μl of forward and reverse primers, 8.2 μl of DNase and RNase-free water, and 10 μl of Applied Biosystems^®^ SYBR^®^ Green PCR Master Mix (Applied Biosystems) was performed by using 7500 Fast Real-time PCR System (Applied Biosystems). Sequences for primers are provided in **Supplementary Table S2**.

### Immunohistochemistry

Upon dehydration by sequential soaking in 20% and 30% sucrose solution, 18 μm serial coronal section of the substantia nigra was prepared by using a cryostat (Leica, Wetzlar, Germany). The sections were then boiled in citrate buffer for 15 min using a microwave for antigen retrieval, followed by removal of endogenous peroxidases in 0.3% H_2_O_2_-methanol (v/v) for 15 min at room temperature. Nonspecific binding was blocked with 3% donkey serum for 30 min at room temperature. The slides were then incubated with polyclonal rabbit anti-tyrosine hydroxylase (TH) antibody (1:4,000, AB152, Sigma-Aldrich, St. Louis, MO, USA) overnight at 4°C, followed by washing with PBS and incubation with goat anti-rabbit IgG at room temperature for 1 h and subsequently in streptavidin peroxidase for 30 min at 37°C. TH-positive neurons were visualized by diaminobenzidine (DAB). Images of TH-positive cells were observed under ×100 magnification and captured using a photoscope (Olympus BX51, Olympus Corporation, Tokyo, Japan). The average number of TH-positive neurons from four fields of view was compared between groups.

### Preparation of RD-Treated Serum

Young adult OVX rats were given vehicle or RD at 2.0 g/kg day (10 times of its clinical equivalent dose) for 3 consecutive days and pair-fed with phytoestrogen-free AIN-93 diet. Upon the last oral administration on day 3, the rats were fasted overnight and given drugs one more time in the following morning. Rats were euthanized an hour after administration and blood samples were collected. Serum was prepared upon centrifugation and stored at −80°C. LC-MS analysis of the serum was performed to confirm the presence of a major chemical marker from RD. Methanol extract of serum was prepared, and extract of 1 ml serum was dissolved in 1 ml of ethanol; the concentration of this solution was defined as “1.” Microsep™ Advance Centrifugal Device (3K, PALL. Cor., Port Washington, NY, USA) was used to remove small molecules including steroid in serum extract, and the solution was sterilized with a 0.22-µm membrane. Final dilution of the serum extract (10^−5^, 10^−4^, 10^−3^, and 10^−2^) was used for cell studies.

### Cell Culture and Measurement

Four ER-positive cell lines, including human breast MCF-7 cells (ATCC^®^ HTB-22™, passage 8-15), endometrial Ishikawa cells (kindly provided by Dr. Lihui Wei at Peking University People’s Hospital, passage 12-18), neuroblastoma SHSY5Y cells (ATCC^®^CRL-2266™, kindly provided by Prof. Wenfang Chen at Qingdao University, passage 15-25), and osteosarcoma MG-63 cell (ATCC^®^ CRL-1427™, passage 3-10) were routinely cultured according to ATCC (Gaithersburg, MD, USA) instruction. Cells were seeded in 96-well or 24-well plate at a density of 0.8 × 10^3^ and 2.0 × 10^4^/well, respectively, for different assays. The medium was changed to phenol red-free medium containing charcoal-stripped FBS for another 24 h. Cells were treated with extract of serum treated with vehicle or RD at various dilutions (10^−5^, 10^−4^, 10^−3^, and 10^−2^), tamoxifen (10^−12^ to 10^−6^ M) and their combinations at optimal concentrations for 48 h. Cell viability or ALP activity were measured by MTS assay or ALP assay, respectively. Briefly, upon treatment, medium was discarded and replaced with 100 µl of MTS/phenazine methosulfate working solution (Promega, Madison, WI, USA). Absorbance at 490 nm was measured with a microplate reader (CLARIOstar, BMG LABTECH, Ortenberg, Germany) after incubation at 37°C for 1 to 4 h. Relative absorbance to control group was analyzed and compared between treatment groups and controls. For ALP assay, upon treatment, 100 µl of passive lysis buffer (PLB) was added to each well to lyse cell. The ALP activity of cell lysate was measured by a LabAssayTM ALP Kit (Wako, Japan) following manufacturer’s instruction. Total protein concentrations of the cell lysate were measured *via* Bradford method to normalize ALP activity.

### Statistical Analysis

Data were expressed as mean ± SEM. The differences between groups of *in vivo* study were analyzed by one-way ANOVA with Tukey’s *post-hoc* test. The differences between treatment groups and control in *in vitro* study were determined by Independent *t*-test. Two-way ANOVA with Bonferroni as *post-hoc* test was performed to analyze the interaction between RD and tamoxifen. A *p*-value <0.05 was considered statistically significant.

## Results

### Herbal Extract Preparation and Authentication

HPLC assay confirmed that the quality of *rhizoma drynariae* raw herb has fulfilled the requirements on the presence of naringin (**Supplementary Data 3A**). The amount of naringin in RD extract was 0.394 mg/g. The datasets presented in this study can be found in online repositories. The names of the repository/repositories and accession number(s) can be found below: (Figshare https://doi.org/10.6084/m9.figshare.17084834.v5)

### Estrogenic Activities of RD and Its Combination With Tamoxifen in Mature OVX Rats

**Figure 1:**
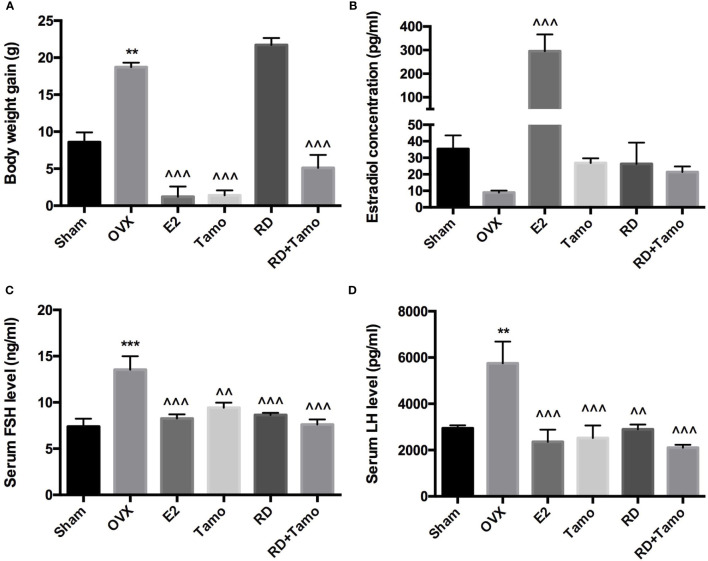
Estrogenic effects of RD, tamoxifen, and their combinations on body weight gain and circulating hormone level in mature ovariectomized rats. Six-month-old mature Sprague Dawley sham-operated (Sham) or ovariectomized (OVX) rats were treated with either vehicle, E2 (1.0 mg/kg/day), RD (0.2 g/kg/day), tamoxifen (Tamo, 0.1 mg/kg/day), or their combination for 12 weeks. **(A)** Body weight gain of OVX rats after 12-week treatments. **(B–D)** Circulating level of estradiol, FSH, and LH was measured by using EIA kit (CayMan). Data were expressed as mean ± SEM. *n* = 6 to 10. ^***^p < 0.001, ^**^p < 0.01 vs. Sham; ^^^^*p* < 0.01, ^^^^^*p* < 0.001 vs. OVX. *p*-value of two-way ANOVA: 0.7253 for body weight, 0.1104 for estradiol concentration, 0.0239 for serum FSH, and 0.0331 for serum LH.

In response to OVX, a significant increase in body weight was observed in OVX rats in comparison with Sham rat (*p* < 0.01 vs. Sham), which was reversed by the treatments with E2, tamoxifen, or cotreatment of tamoxifen and RD (*p* < 0.001, vs. OVX) but not RD alone (**Figure 1A**). Cotreatment with RD attenuated the inhibitory effects of tamoxifen on body weight gain. Results of two-way ANOVA indicated that RD did not interact with tamoxifen but decreased the inhibitory effect of tamoxifen on body weight gain without significance (RD × Tamo: *p* = 0.7253).

Decreased estrogen production was believed to be the sole reason for menopausal symptoms. However, recent findings suggest that dramatic increase in FSH appears much earlier than the drop of estrogen before the onset of menopause and accounts for the appearance of menopausal symptoms (10). The results of the present study showed that the dramatic decrease in serum estradiol in OVX rats was accompanied with the significant increase in circulating levels of FSH and LH (**Figures 1B–D**, *p* *<* 0.01 vs. Sham). As expected, the treatment of OVX rats with estradiol significantly reversed serum level of estradiol and completely suppressed FSH and LH levels to comparable level with those of Sham rats. The increases in serum estradiol were also observed in OVX rats treated with tamoxifen, RD, as well as their combinations while the changes did not reach statistical significance (**Figure 1B**, vs. OVX). Similar to estrogen, tamoxifen, RD alone, and their combination all dramatically suppressed the increase in both circulating FSH and LH level in OVX rats (**Figures 1C, D**, *p* < 0.01 vs. OVX). No statistical difference in reproductive hormone level was detected between OVX rats treated with tamoxifen and OVX rats treated with its combination with RD, indicating RD did not alter actions of tamoxifen on reproductive hormones. The two-way ANOVA analysis indicated the interactions between RD and SERMs on inhibiting FSH and LH levels (RD × tamoxifen, FSH: *p* = 0.0239, LH: *p* = 0.0331) but not on restoring estradiol level, in OVX rats.

**Figure 2:**
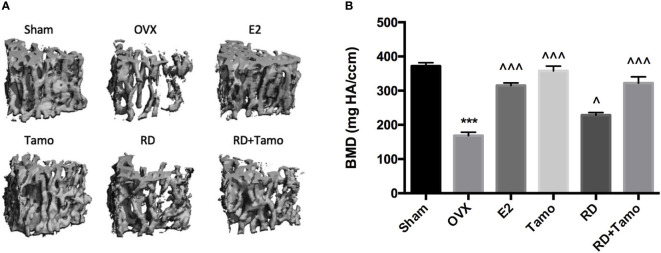
Estrogenic effects of RD, tamoxifen, and their combinations on bone microarchitecture and bone mineral density of the lumbar spine in mature ovariectomized rats. Upon treatment, lumbar vertebra was collected. Bone microarchitecture **(A)** and bone mineral density (BMD) of the lumbar vertebra **(B)** were measured by micro-CT. Data were expressed as mean ± SEM. *n* = 6 to 10. ^***^p < 0.001 vs. Sham; ^*p* < 0.05, ^^^^^*p* < 0.001 vs. OVX. *p*-value of two-way ANOVA: 0.0013 for BMD.

In response to estrogen deficiency and probable increased FSH, OVX rats experienced severe bone loss as indicated by the deteriorated bone structure (**Figure 2A**), the decreased BMD (**Figure 2B**, *p* < 0.001), and degradation in trabecular bone properties, such as the decreases in BV/TV, Tb.N, Conn.D, and Tb.Th, as well as the increase in Tb.Sp (**Table 1**, *p* < 0.001 vs. Sham) at the lumbar vertebra. Estrogen or tamoxifen alone exerted potent antiosteoprotective activity and attenuated OVX-induced bone loss as expected (**Figures 2A, B** and **Table 1**, *p* < 0.001 vs. OVX). RD alone and in combination with tamoxifen significantly restored OVX-induced changes in bone structure, BMD, and bone properties (*p* < 0.001 vs. OVX). The result of two-way ANOVA confirmed the interaction between tamoxifen and RD on promoting BMD (RD × Tamo: *p* = 0.0013) and improving trabecular bone properties (RD × Tamo: *p* = 0.0014 for BV/TV, *p* = 0.0002 for Conn.D, *p* = 0.0049 for Tb.N, *p* = 0.0022 for Tb.Th, *p* = 0.0017 for Tb.Sp) in OVX rats. Despite the interactions, RD did not induce alteration in the stimulatory activities of tamoxifen on BV/TV, Tb.N, and Tb.Th and inhibitory effect on Tb.Sp.

**Table 1:** The effect of RD, tamoxifen, or their combination on trabecular bone properties and bone turnover markers in mature ovariectomized rats.

	Lumbar Vertebra					Turnover Biomarkers	
	BV/TV	Conn.D	Tb.N (1/mm)	Tb.Th (mm)	Tb.Sp (mm)	OCN (ng/ml)	DPD (nmol/mmol)
Sham	0.452 ± 0.009	33.97 ± 2.70	3.50 ± 0.06	0.129 ± 0.003	0.245 ± 0.005	11.4 ± 0.419	224.0 ± 16.4
OVX	0.117 ± 0.006^***^	9.57 ± 1.47^***^	1.91 ± 0.04^***^	0.087 ± 0.003^***^	0.495 ± 0.015^***^	28.9 ± 1.291^***^	670.1 ± 98.7^***^
E2	0.379 ± 0.027^^^^^	25.70 ± 2.34^^^^^	3.36 ± 0.06^^^^^	0.123 ± 0.008^^^^^	0.295 ± 0.025^^^^^	14.6 ± 0.790^^^^^	370.4 ± 32.9^^^^^
Tamo	0.409 ± 0.023^^^^^	33.00 ± 2.30^^^^^	3.32 ± 0.09^^^^^	0.135 ± 0.005^^^^^	0.266 ± 0.011^^^^^	15.9 ± 0.591^^^^^	463.9 ± 23.7^
RD	0.199 ± 0.010^^^	18.99 ± 1.10^^^	2.38 ± 0.09^^^	0.105 ± 0.002^^^	0.407 ± 0.015^^^^	23.8 ± 0.789^^^^^	484.5 ± 37.3
RD+Tamo	0.354 ± 0.029^^^^^	25.78 ± 2.04^^^^^	3.10 ± 0.13^^^^^	0.127 ± 0.004^^^^^	0.288 ± 0.016^^^^^	15.4 ± 0.441^^^^^	412.5 ± 26.9^^^^
*p* value of Two-way ANOVA							
RD × Tamo	0.0014	0.0002	0.0049	0.0022	0.0017	0.0128	0.1974

BV/TV, bone volume over total volume; DPD, deoxypyridinoline; Conn.D, connectivity density; OCN, osteocalcin; Tb.Sp, trabecular bone separation; Tb.Th, trabecular bone thickness; Tb.N, trabecular bone number. n = 6 to 12. ^**^p < 0.01, ^***^p < 0.001 vs. Sham; ^^^^p < 0.01, ^^^^^p < 0.001 vs. OVX.

Serum OCN is an osteoblast-produced biomarker for bone formation while urinary DPD, generated from the breakdown, is a biomarker for bone resorption (11). Serum OCN (28.9 ± 1.291 ng/ml) and urinary DPD (670.1 ± 98.7 nmol/mmol) were significantly increased in OVX rats (**Table 1**, *p* < 0.001 vs. Sham). All treatments exhibited inhibitory effects on the increases in bone turnover biomarkers (**Table 1**, *p* < 0.05 vs. OVX), suggesting their bone protective actions might be mediated by suppressing bone turnover. RD interacted with tamoxifen on decreasing serum level of OCN (RD × Tamo: *p* = 0.0128) but not on urinary DPD (RD × Tamo: *p* = 0.1974). However, RD did not alter the responses of either OCN or DPD to treatment with tamoxifen in OVX rats (SERMs alone vs. TCMs+SERMs).

**Figure 3:**
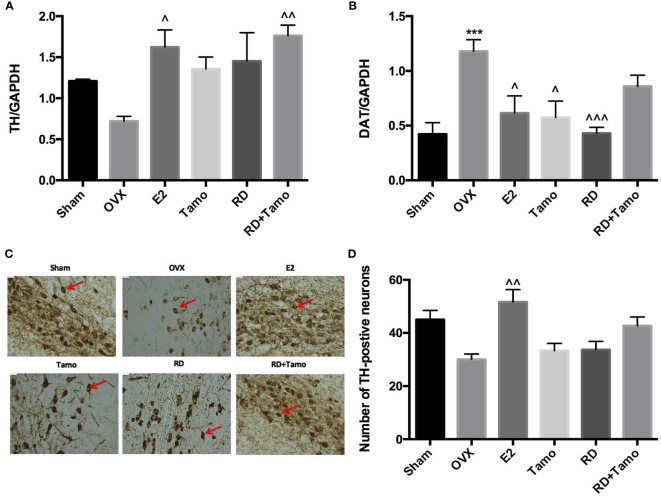
Estrogenic effects of RD, tamoxifen, and their combinations in the central nervous system of mature ovariectomized rats. Upon treatment, striatum was collected at euthanasia. mRNA expression of *tyrosine hydroxylase* (*TH*) **(A)** and *dopamine transporter* (*DAT*) **(B)** were measured by real-time PCR. TH-positive cells in the substantia nigra were visualized by IHC (×100) **(C)** and quantified **(D)**. Data were expressed as mean ± SEM. *n* = 6 to 10. ^***^p <0.001 vs. Sham; ^^^*p* < 0.05, ^^^^*p* < 0.01, ^^^^^*p* < 0.001 vs. OVX. *p*-value of two-way ANOVA: 0.4257 for TH/GAPDH, <0.0001 for DAT/GAPDH, 0.35531 for TH-positive neurons.

To investigate if RD also exerted estrogenic activities in the central nervous system, the mRNA expressions of *TH* (12), *dopamine transporter* (*DAT*) in the striatum and TH-positive neurons in the substantia nigra were measured by real-time PCR and immunohistochemistry (IHC), respectively. Six months upon OVX, *TH* mRNA (**Figure 3A**) was downregulated while the mRNA expression of *DAT* (**Figure 3B**, *p*<0.001 vs. Sham) was noticeably upregulated in the striatum in OVX rats. IHC result confirmed the downregulated expression of TH as advised by the decreased numbers of TH-positive neurons in the substantia nigra of OVX rats (**Figures 3C, D****)**. The treatments with E2, tamoxifen, or RD alone, as well as their combination produced pronounced increase in *TH* mRNA expression and decrease in *DAT* mRNA expression (**Figures 3A, B**, *p* < 0.05). RD, tamoxifen, or their combination appeared to stimulate the TH-positive neurons while changes did not reach a statistical significance (**Figures 3C, D****)**. These results suggested the potential valuable effects of RD in the CNS. The two-way ANOVA results demonstrated that RD interacted with tamoxifen to suppress *DAT* mRNA expression in the striatum of OVX rats (RD × Tamo: *p* < 0.0001) without affecting TH expression (RD × Tamo: *p* = 0.4257 for *TH* mRNA, *p* = 0.3551 for TH-positive neuron number).

**Figure 4:**
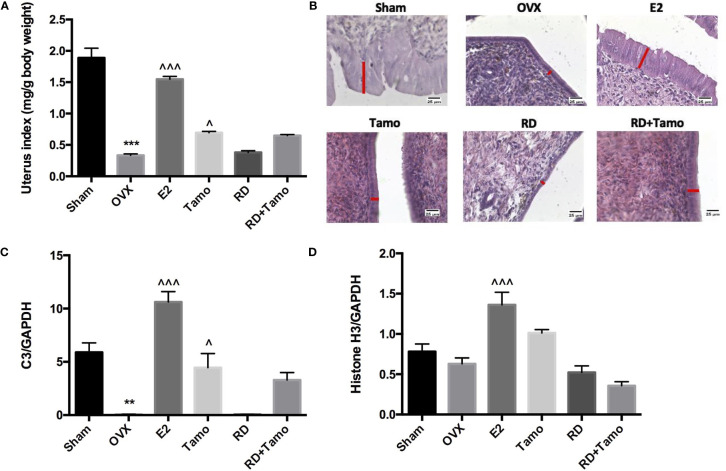
Estrogenic effects of RD, tamoxifen, and their combinations on uterine index, mRNA expression of estrogen-responsive gene, and endometrial morphology in mature ovariectomized rats. Upon treatment, uterus was collected and weighed at euthanasia. Ratio of the uterus weight to body weight was recorded as uterine index (mg/g) and compared between groups **(A)**. Morphology of endometrium (×400, thickness of endometrium was indicated by the length of the red line) was visualized by H&E staining **(B)**. mRNA expression of *complement component 3* (*C3*) and *histone H3* were measured by real-time PCR **(C, D)**. Data were expressed as mean ± SEM. *n* = 6 to 10. ^**^p < 0.01, ^***^p < 0.001 vs. Sham; ^^^*p* < 0.05, ^^^^^*p* < 0.001 vs. OVX. *p*-value of two-way ANOVA: 0.0847 for uterine index, 0.4800 for C3/GAPDH, and 0.0003 for histone H3/GAPDH.

**Figure 5:**
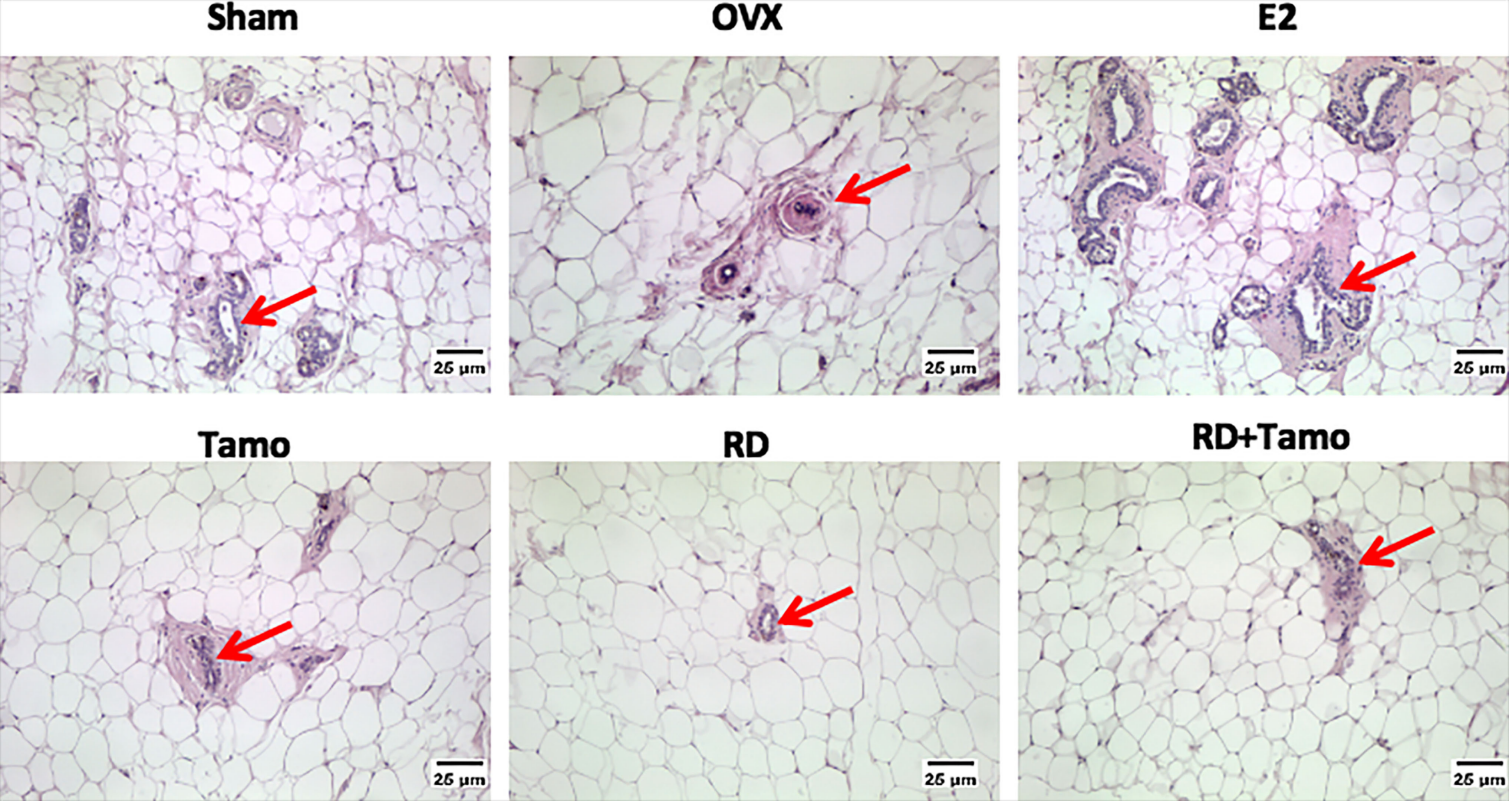
Estrogenic effects of RD, tamoxifen, and their combinations on the morphology of breast tissue in mature ovariectomized rats. Upon treatment, breast tissue was collected in 4% paraformaldehyde and the morphology of mammary gland (as indicated by the red arrow, ×100) was visualized by H&E staining.

In response to OVX, uterine weight of OVX rats was significantly decreased to about 25% of Sham rats (**Figure 4A**, *p* < 0.001 vs. Sham) and a dramatic atrophy was observed in the uterus of OVX rats as revealed by the shrunken endometrium (**Figure 4B**, indicated by the red line). The treatment with E2, tamoxifen alone, or its combinations with RD, but not RD alone, significantly increased the weight of the uterus in OVX rats (*p* < 0.05 vs. OVX rats), confirming the uterotrophic effects of tamoxifen. The changes in weight of the uterus in response to different treatments were in consistence with the increase in the thickness of the endometrium (**Figure 4B**). Similar to estradiol, tamoxifen alone or its combination with RD, but not RD alone, stimulated the mRNA expression of *complement component 3* (*C3*, **Figure 4C**, *p* < 0.05) and *histone H3* (*HH3*, **Figure 4D**, *p* < 0.001), two estrogen-responsive genes, in the uterus of OVX rats. The two-way ANOVA results suggested the interaction between RD and tamoxifen on stimulating *HH3* mRNA expression (RD × Tamo: *p* = 0.0003). Ovariectomy also induced atrophy of breast tissue as indicated by the reduced number of mammary ducts in OVX rats, which was reversed by estrogen (**Figure 5**). Tamoxifen enhanced the atrophy in the mammary gland of OVX rats (**Figure 5**). Unlike either estrogen or tamoxifen, RD did not induce any growth of the mammary duct in OVX rats alone or in combination with tamoxifen. Our results suggest that RD did not induce estrogenic effects in either the uterus or mammary gland in OVX rats while tamoxifen promoted the growth of the endometrium.

### Direct Estrogenic Activities of DBT and Combination With Tamoxifen *In Vitro*

**Figure 6:**
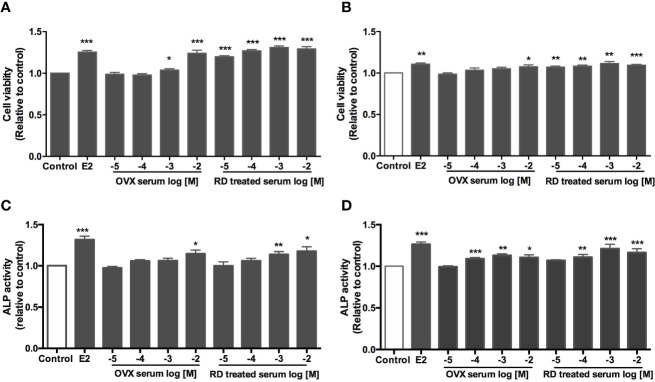
Direct estrogenic effects of OVX serum or RD-treated serum in ER-positive cells. Human breast cancer MCF-7 **(A)** neuroblastoma SHSY5Y **(B)** endometrial cancer Ishikawa **(C)** and osteosarcoma MG-63 cells **(D)** were treated with E2 (10 nM), OVX serum (10^−5^–10^−2^ dilution) or RD-treated serum (10^−5^–10^−2^ dilution) for 48 h. MTS assays were performed in MCF-7 and SH-SY5Y cells while ALP activities were measured in MG-63 cells and Ishikawa cells. Results were expressed as ratio to control. *n* = 3 or more. ^*^p < 0.05, ^**^p < 0.01, ^***^p < 0.001 vs. control.

**Figure 7:**
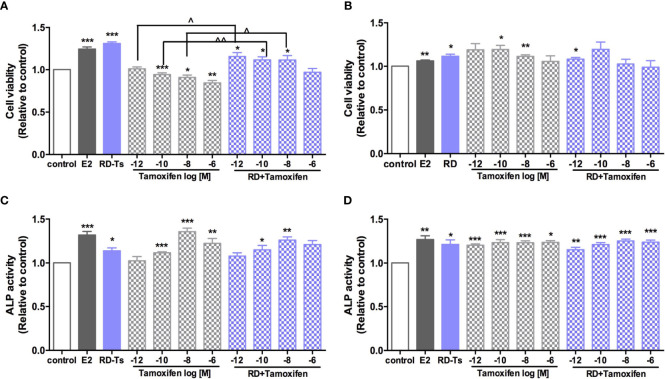
Direct estrogenic effects of RD-treated serum, tamoxifen, and their combinations in ER-positive cells. Human breast cancer MCF-7 **(A)**, neuroblastoma SHSY5Y **(B)**, endometrial cancer Ishikawa **(C)**, and osteosarcoma MG-63 cells **(D)** were treated with E2 (10 nM), tamoxifen (10^−12^–10^−6^ M) or RD-treated serum (10^−3^ dilution) for 48 h. MTS assays were performed in MCF-7 and SH-SY5Y cells while ALP activities were measured in MG-63 cells and Ishikawa cells. Results were expressed as ratio to control. *n* = 3 or more. ^*^p < 0.05, ^**^p < 0.01, ^***^p < 0.001 vs. control; ^^^*p* < 0.05, ^^^^*p* < 0.01 vs. tamoxifen alone. *p*-value for interaction between RD and tamoxifen at 10^−12^, 10^−10^, 10^−8^, and 10^−6^ M of two-way ANOVA: 0.0045, 0.0089, 0.0728, and 0.0028 for cell viability in MCF-7 cell; 0.0141, 0.2393, 0.0068, and 0.0816 for cell viability in SH-SY5Y cell; 0.2598, 0.1249, 0.0059, and 0.1098 for ALP activity in Ishikawa cell; and 0.1080, 0.258, 0.0222, and 0.5987 for ALP activity in MG-63 cell.

Four ER-positive cell lines in line with the four estrogen-sensitive tissues characterized in the OVX rats, including human breast cancer MCF-7 cells, endometrial cancer Ishikawa cells, neuroblastoma SHSY5Y cells, as well as osteosarcoma MG-63 cells, were employed to investigate the estrogenic activities of RD *in vitro*. The results of LC-MS confirmed the presence of naringin and naringenin 5,7-di-*O*-glucoside in RD-treated serum extract, suggesting that RD and its metabolites were absorbed and transported in rat circulation (**Supplementary Data 3B**). The datasets presented in this study can be found in online repositories. The names of the repository/repositories and accession number(s) can be found below: (Figshare https://doi.org/10.6084/m9.figshare.17084834.v5). Compared with the extract of the vehicle-treated serum, RD-treated serum extract dose-dependently increased cell viability [**Figure 6A** (MCF-7), **B** (SH-SY5Y)] and/or ALP activity [**Figure 6C** (Ishikawa), **Figure 6D** (MG-63)], of which RD exerted the most potent effects at 10^−3^ dilution. Tamoxifen exerted inhibitory effect on MCF-7 cell proliferation (**Figure 7A**, *p* < 0.05) but stimulatory effects on SHSY5Y cell proliferation (**Figure 7B**, *p* < 0.05) as well as ALP activity in Ishikawa (**Figure 7C**, *p* < 0.05) and MG-63 cells (**Figure 7D**, *p* < 0.05). The two-way ANOVA results suggested that RD at 10^−3^ dilution interacted with tamoxifen at certain concentrations in all the four cell lines (**Figures 7A–D**, RD × Tamo: *p* < 0.05). RD markedly reversed the inhibitory effects of tamoxifen at 10^−12^, 10^−10^, and 10^−8^ M on cell proliferation in MCF-7 cells (**Figure 7A**, *p* < 0.05) but does not affect the actions of tamoxifen in either SHSY5Y, Ishikawa, or MG-63 cells.

## Discussion

The use of “kidney-tonifying” herb, RD, is an alternative to SERMs or HRT in managing osteoporosis in China. Our previous studies demonstrated the estrogenic effect of RD in promoting bone health in OVX rats. The present study further addressed the in vivo and in vitro drug-herb interactions of RD at its clinical dose with SERMs in four estrogen-sensitive tissues, including bone, brain, uterus and breast, what are one of the major clinical concerns when patients are cotreated with them. Our results also showed that RD did interact with SERMs but did not alter the action of tamoxifen in all tissues. Unlike tamoxifen, RD alone exerted estrogenic effects in tissue-selective manner, by which RD did not cause side effects on the uterus and breast but has stimulatory impact in the bone and brain.

Dramatic changes in hormonal secretions, including estradiol, FSH, and LH, during menopause cause various negative physiological consequences, including breast, uterus, skeletal, and neuronal tissues (13). The present *in vivo* study showed that OVX in rats caused a remarkable reduction in serum estradiol level but an induction in serum FHS and LH levels. RD treatment or cotreatment of RD with tamoxifen could restore both FSH and LH levels but not E2. It indicated that RD and SERMs might modulate sex hormone level and exert hormonal effect on breast, uterus, skeletal, and neuronal tissues. Further study is needed to confirm if RD has any impact on hypothalamus-pituitary-gonad axis which is the major signaling pathway controlling the releases of sex hormone.

Postmenopausal osteoporosis is the second cause of osteoporosis in women. OVX model in rats mimic the metabolic modification related to deficiency of estradiol in women. Our results demonstrated a significant reduction in serum estradiol level and uterus weight in OVX rats. Such changes were accompanied with bone loss in the lumbar spine in rats. As expected, RD and tamoxifen, like E2, were effective in attenuating bone loss at three bone sites by increasing BMD and improving bone microarchitecture which are in line with our previous studies (7, 9). Also, RD significantly restored serum OCN level, an osteoblastic biomarker in bone remodeling (14), while tamoxifen restored both serum OCN and DPD levels, a collagen breakdown biomarker during bone resorption (9) in OVX rats. These results suggested the estrogen-like effect of RD and tamoxifen in osteoporotic rats, in which RD acted mainly on bone formation while tamoxifen governed both bone formation and resorption. Also, our previous study demonstrated a positive correlation between BMD and serum E2 and a negative correlation between BMD and serum FSH or LH at the distal femur in OVX rats (15). FSH has also been reported to regulate osteoclastogenesis, bone resorption, and bone turnover biomarkers in postmenopausal women (16, 17). Thus, our results suggested that the bone protective effects of RD and tamoxifen might be mediated by the modulation of sex hormone level.

Epidemiological evidence suggests postmenopausal women are at high risk of Parkinson’s disease. Circulating estradiol level was reported to act as a neuroprotective agent on the dopaminergic system in women (18, 19). In the present study, OVX in rats was shown to affect the TH level for dopamine production and higher DAT level for dopamine resorption in the striatum. The increases in DAT in OVX rats could be reversed by the treatment of RD or tamoxifen alone, indicating their neuroprotective effects. These are in consistence with other studies indicating that tamoxifen directly interacted with the DAT to prevent dopamine uptake and blocked dopamine efflux in rats (20). Also, the hypothalamic dysfunction in PD patients could lead to the disruption of pituitary hormone secretion (FSH, LH, and testosterone) and dopamine content (21). Therefore, the reclamation of serum FSH or LH level in OVX rats after RD or tamoxifen treatment might also be a sign of their neuroprotective effects on the hypothalamus.

Estrogenic side effects on reproductive organs, like the breast and uterus, are always the main concerns when using phytoestrogen-containing drug or SERMs because they could stimulate the growth of reproductive tissues through estrogen receptors (22–24). In the present study, tamoxifen treatment did exert stimulatory effects on endometrium layer and uterus weight and estrogen-responsive gene but not in breast tissues. These results are comparable with those of others that tamoxifen acts as an ER antagonist in breast and is clinically prescribed for the treatment of ER-positive breast cancer (2). However, it was shown to elevate the risk of endometrium cancer in women (25). In contrast to tamoxifen, although RD contains flavonoid, naringin being known as a phytoestrogen, which was reported to act on estrogen receptors (26), RD treatment did not show any stimulatory effect in both the breast and uterus in OVX rats in the present study. It advocated the use of RD as its clinical dose was safer than tamoxifen in OVX rats. Our results suggest that RD might serve as an alternative SERM that could selectively exert agonistic estrogenic protection in the bone and brain and antagonist action in reproductive organs.

Systemic activation of TCM after administration *via* metabolism produces metabolites, which are usually considered to be the bioactive component of the herbal medicine in the body. Thus, the direct effect of RD-treated serum was evaluated in estrogen-sensitive cell lines from four tissues. The results indicated that RD-treated serum, acting like E2 but weaker, promoted the cell viability of MCF-7 cells and SH-SY5Y cells as well as the ALP activity in MG-63 cells and Ishikawa cells. However, these seem to be in contrast with the *in vivo* results that RD did not stimulate reproductive tissues. The discrepancy between *in vitro* and *in vivo* might be due to the differences in the dosages of RD used in cells and animals. The effects of phytoestrogen has been demonstrated to be either agonistic or antagonistic depending on its own concentrations and the estrogen concentrations of the environment (27). For the *in vivo* study, the dosage of RD used was converted from its clinical dosage and the level of phytoestrogens and their metabolites in RD is not high enough to compete with SERMs to bind with ERs. In contrast, RD-treated serum used in the *in vitro* studies was prepared by treatment of OVX rats with high dosage of RD (10 times to its *in vivo* dose), potentially enabling the major phytoestrogen components and main metabolites in RD-treated serum to reach the level to activate ERs and induce stimulatory effects in Ishikawa and MCF-7 cells.

With the increased use of TCM worldwide, the concern on drug–herb interaction is exaggerating, especially when both drug and herb target estrogen receptors. Our two-way ANOVA analysis showed that RD could interact with tamoxifen in promoting bone health, exerting neuroprotection, increasing uterine index in OVX rats, and stimulating ALP or cell viability in cells. However, cotreatment with RD did not significantly alter the responses to tamoxifen in estrogen-sensitive tissues. It might be due to their weak binding affinity to ERs compared with tamoxifen. Further study is needed to compare the binding affinity of tamoxifen and major flavonoid as well as their metabolites in RD.

To the best of our knowledge, this is the first investigation on the drug–herb interaction between RD and tamoxifen in OVX model and using biologically activated RD in four ER-positive human cell lines. We confirmed that RD could tissue-selectively exert estrogenic protection in the bone and neuron without causing side effects on the breast and uterus, which differentiates RD from tamoxifen. The drug–herb interaction of RD and tamoxifen did not weaken or strengthen the action of tamoxifen in all tissues, suggesting that RD alone and in combination with tamoxifen might be considered effective and safe alternative methods for the management of menopause-related symptoms. However, further clinical investigation should be made to confirm the preclinical observations in the present study.

## Data Availability Statement

The datasets presented in this study can be found in online repositories. The names of the repository/repositories and accession number(s) can be found below: https://doi.org/10.6084/m9.figshare.17084834.v5

## Ethics Statement

The animal study was reviewed and approved by the Hong Kong Polytechnic University Animal Subjects Ethics Sub-committee (ASESC Case: 15-16/31-ABCT-R122 HMRF).

## Author Contributions

LZ and K-YW are the major contributors of the present study, performing experiments, analyzing the data, and preparing the manuscript. WY, CP, and HX helped in performing and sample collection of animal experiments. C-OC and DM performed the chemical analysis of the RD extract. M-SW conceived and supervised the experiments and finalized the manuscript. All authors listed have made a substantial, direct, and intellectual contribution to the work and approved it for publication.

## Funding

This work was supported by the Health and Medical Research Fund (HMRF) grant (Ref. No. 13143771) of Hong Kong.

## Conflict of Interest

The authors declare that the research was conducted in the absence of any commercial or financial relationships that could be construed as a potential conflict of interest.

## Publisher’s Note

All claims expressed in this article are solely those of the authors and do not necessarily represent those of their affiliated organizations, or those of the publisher, the editors and the reviewers. Any product that may be evaluated in this article, or claim that may be made by its manufacturer, is not guaranteed or endorsed by the publisher.

## Abbreviations

BMD, bone mineral density; BV/TV, bone volume over total volume; DAT, dopamine transporter; DPD, deoxypyridinoline; Conn.D, connectivity density; C3, complement component 3; ER, estrogen receptors; ERE, estrogen response element; FSH, follicle-stimulating hormone; HH3, histone H3; HPG, hypothalamus-pituitary-gonadal; HPLC, high-performance liquid chromatography; HRT, hormone replacement therapy; LC-MS, liquid chromatography-mass spectrometry; LH, luteinizing hormone; OCN, osteocalcin; OVX, ovariectomized; SERMs, selective estrogen receptor modulators; Tb.Sp, trabecular bone separation; Tb.Th, trabecular bone thickness; Tb.N, trabecular bone number; TCMs, traditional Chinese medicine; TH, tyrosine hydroxylase.
