# Supplementary material for: Water Extract of Rhizoma Drynaria Selectively Exerts Estrogenic Activities in Ovariectomized Rats and Estrogen Receptor-Positive Cells
Source: Front Endocrinol (Lausanne). 2022 Feb 24;13:817146. doi: 10.3389/fendo.2022.817146 (PMC8908013; doi:10.3389/fendo.2022.817146)
Supplement: Supplementary file 1 [file DataSheet_1.doc]

Supplementary data

**Supplemental Table 1 Diet composition of AIN-93M diet**

| **Ingredient (g/kg)** | **AIN-93M diet^1^** |
| --- | --- |
| Casein | 140.0 |
| Egg White Solids | 97.9 |
| Methionine | 3.0 |
| Sucrose | 100 |
| Corn Starch | 495.692 |
| Corn Oil | 40.0 |
| Cellulose | 50.0 |
| Mineral mix^2^ (S10022M) | 35 |
| Vitamin mix^3^ (V10037) | 100 |

1. AIN-93M phytoestrogen-free diet is modified with corn oil by Research Diet (D00031602).
2. Mineral mix: Phosphorous (%) 0.23, Potassium (%) 0.38, Sodium (%) 0.11, Magnesium (%) 0.05, Iron (ppm) 34.25, Zinc (ppm) 36.76, Manganese (ppm) 11.09, Copper (ppm) 6.73, Cobalt (ppm) 0.02, Iodine (ppm) 0.21.
3. Vitamin mix: Vitamin A (IU/g) 4, Alpha-Tocopherol (IU/g) 75, Thimine (ppm) 5, Riboflavin (ppm) 6, Niacin (ppm) 30, Pantothenic Acid (ppm)15, Choline (ppm) 1000, Pyrodoxine (ppm) 6, Folic Acid (ppm) 2, Biotin (ppm) 0.2, Vitamin B12 (Mcg/kg) 0.25, Vitamin K (ppm) 0.86.

**Supplementary table 2 Sequences of primers for the estrogen-responsive genes**

| Genes | Primer sequences |
| --- | --- |
| GAPDH | Forward: 5’-TACATTTTGCTGATGACTGG-3’ |
|  | Reverse: 5’-TGAATGGTAGGAGCTTGACT-3’ |
| C3 | Forward: 5’-CTGTACGGCATAGGGATATCACG-3’ |
|  | Reverse: 5’-ATGCTGGCCTGACCTTCAAGA-3’ |
| TH | Forward: 5’-ACACAGCGGAAGAGATTGCT-3’ |
|  | Reverse: 5’-CCCAGAGATGCAAGTCCAAT-3’ |
| DAT | Forward: AAGATCTGCCCTGTCCTGAAAG |
|  | Reverse: CATCGATCCACACAGATGCCTC |

**Supplementary data 3 Chemical analysis of RD raw herb and extract**

**A. HPLC profile**

**
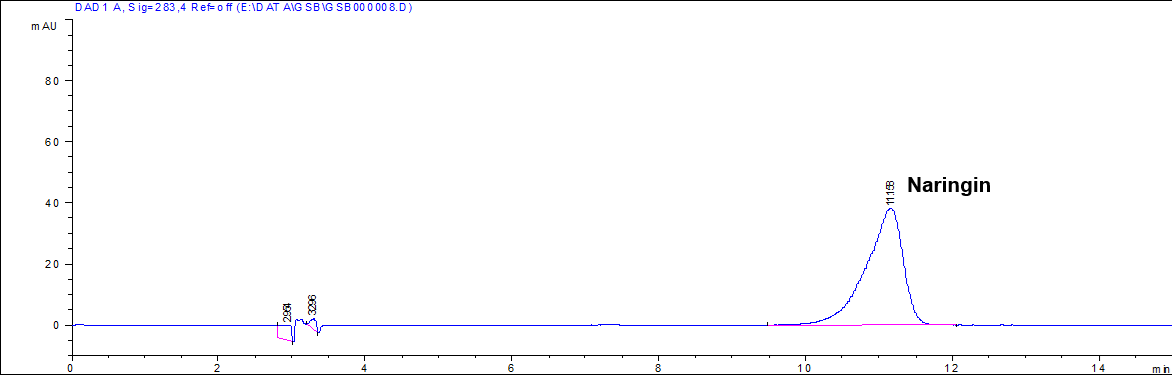
**

**B. LC-MS profile ESI(-) mode**

**
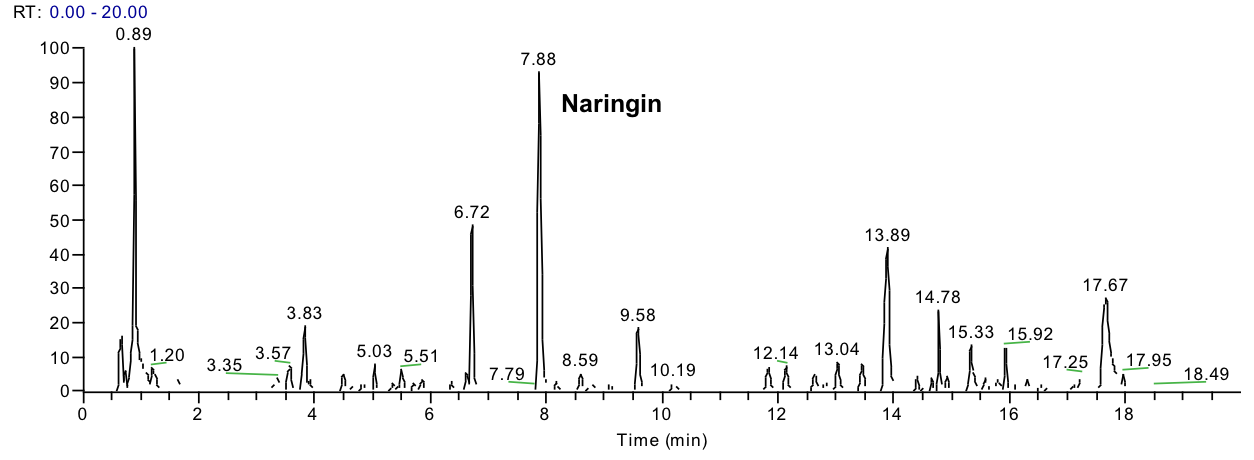
**
